# Supplementary material for: Mechanical Behavior of a Medium-Entropy Fe65(CoNi)25Cr9.5C0.5 Alloy Produced by Selective Laser Melting
Source: Materials (Basel). 2023 Apr 18;16(8):3193. doi: 10.3390/ma16083193 (PMC10145717; doi:10.3390/ma16083193)
Supplement: Supplementary file 1 [file materials-16-03193-s001.zip › materials-2305547-supplementary.pdf]

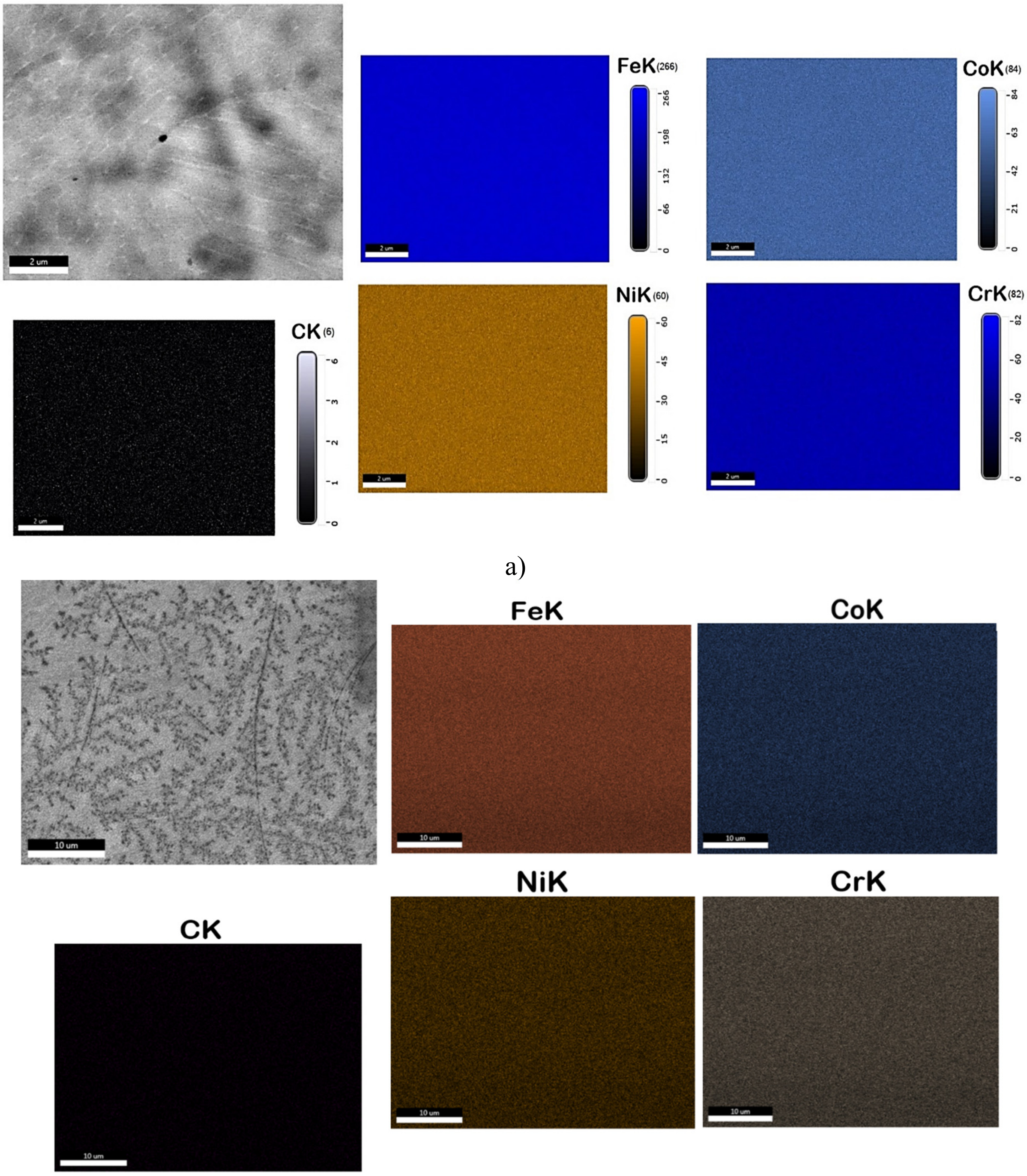

Figure S1. EDS-mapping microstructure of the as - produced (mode: P=200 W and V=1600 mm/s) $Fe_{65}(CoNi)_{25}Cr_{9.5}C_{0.5}$ alloy in the initial (a) and deformed at 77K to ε≈10% (b) conditions. The data obtained suggests chemical homogeneity between the bcc and fcc phases.

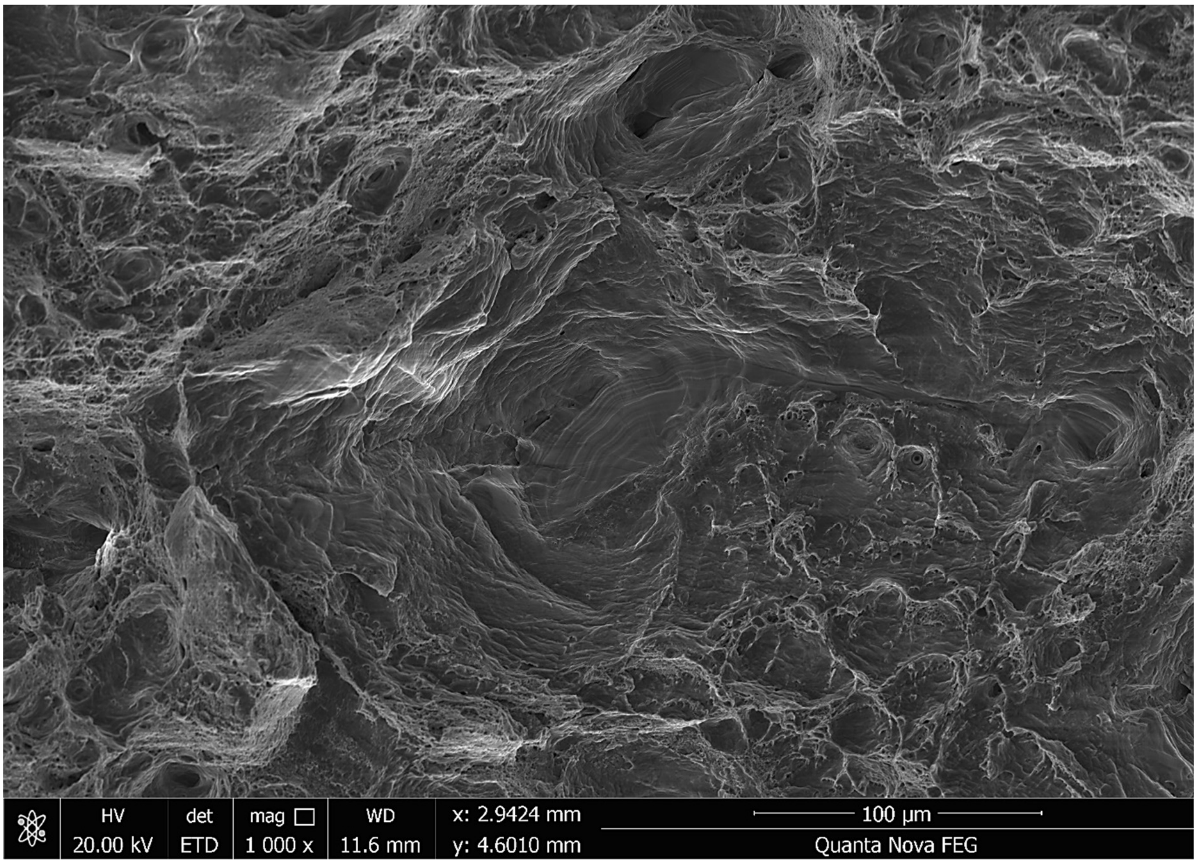

a)

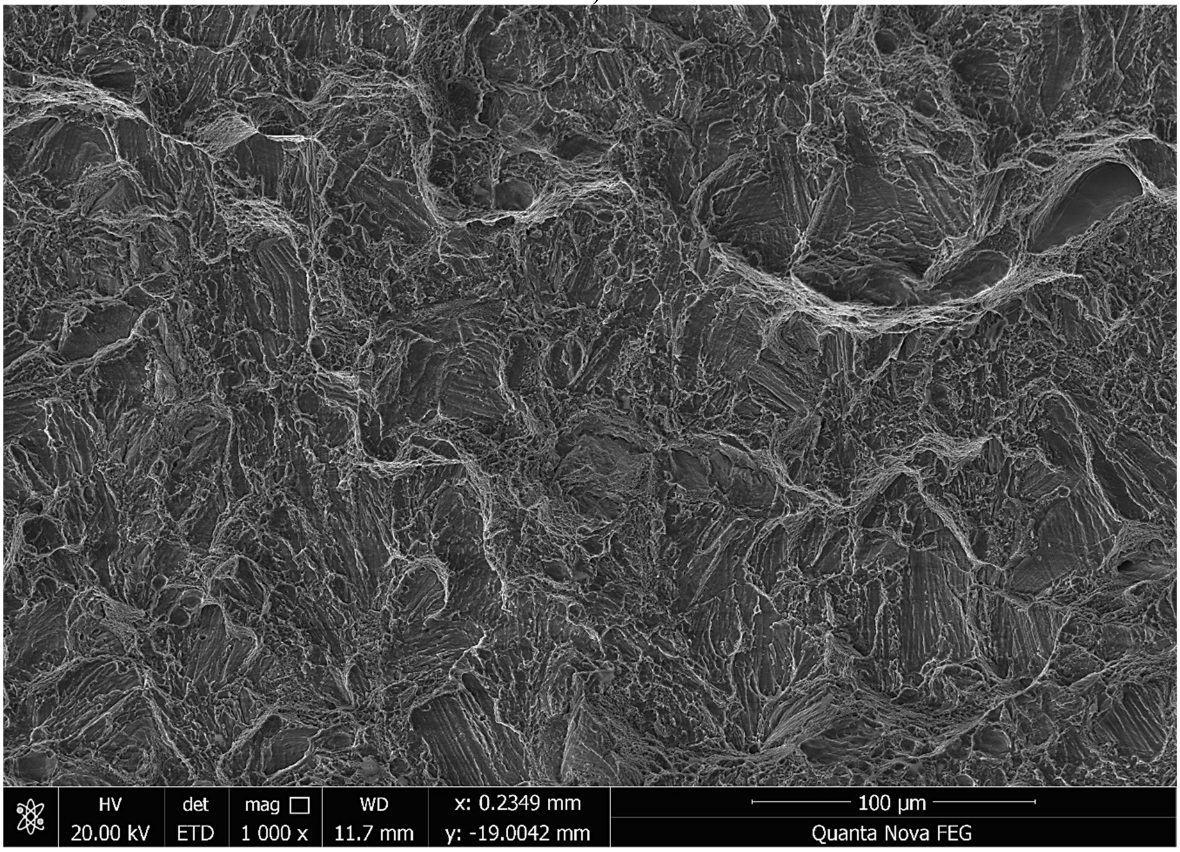

b)

Figure S2. Fractography of specimen fractures after impact toughness tests at room (a) and cryogenic (b) temperatures.

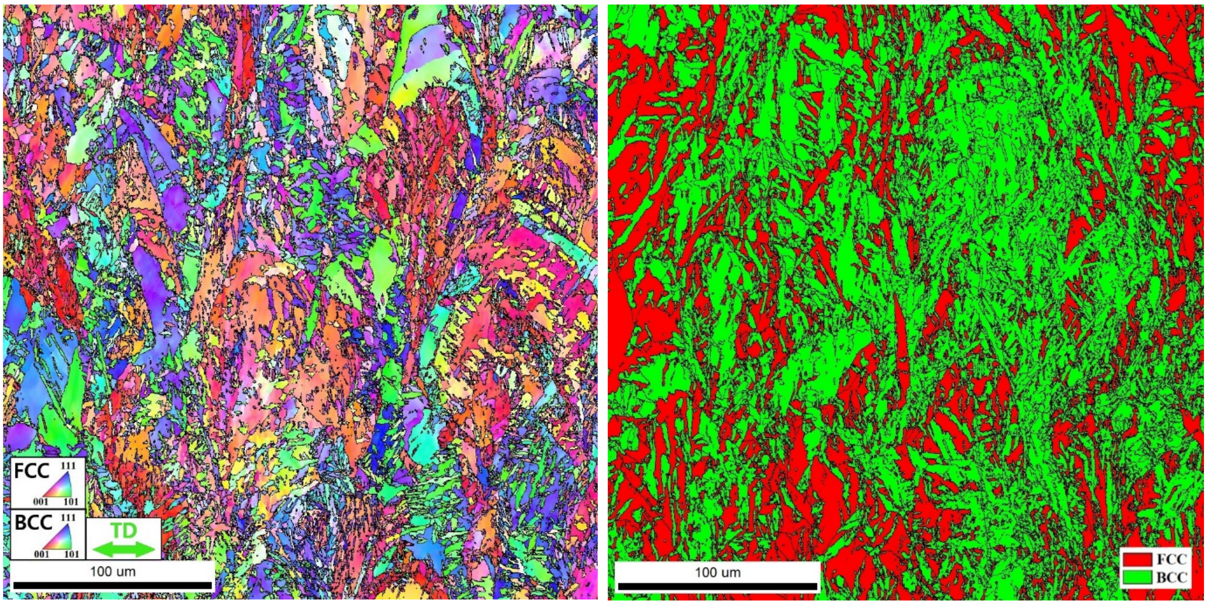

a) b)

Figure S3. IPF (a) and phase (b) maps of the as - produced $Fe_{65}(CoNi)_{25}Cr_{9.5}C_{0.5}$ alloy after interrupted tension to ε=20% at 77 K. Tension of the sample was performed in the horizontal direction relative to the presented image.
